# Microbial communities adhering to the obverse and reverse sides of an oil painting on canvas: identification and evaluation of their biodegradative potential

**DOI:** 10.1007/s10453-012-9281-z

**Published:** 2012-11-16

**Authors:** M. López-Miras, G. Piñar, J. Romero-Noguera, F. C. Bolívar-Galiano, J. Ettenauer, K. Sterflinger, I. Martín-Sánchez

**Affiliations:** 1 Department of Microbiology, Faculty of Sciences, University of Granada, Avda. Fuentenueva, 18071 Granada, Spain; 2 Department of Biotechnology, VIBT-Vienna Institute of Bio Technology, University of Natural Resources and Life Sciences, Muthgasse 11, 1190 Vienna, Austria; 3 Department of Painting, Faculty of Fine Arts, University of Granada, Avda. Andalucía s/n, 18071 Granada, Spain

**Keywords:** Oil painting, Canvas, Biodeterioration, Microbial communities, Spore-forming microorganisms, Enzymatic activities

## Abstract

In this study, we investigated and compared the microbial communities adhering to the obverse and the reverse sides of an oil painting on canvas exhibiting signs of biodeterioration. Samples showing no visible damage were investigated as controls. Air samples were also analysed, in order to investigate the presence of airborne microorganisms suspended in the indoor atmosphere. The diversity of the cultivable microorganisms adhering to the surface was analysed by molecular techniques, such as RAPD analysis and gene sequencing. DGGE fingerprints derived from DNA directly extracted from canvas material in combination with clone libraries and sequencing were used to evaluate the non-cultivable fraction of the microbial communities associated with the material. By using culture-dependent methods, most of the bacterial strains were found to be common airborne, spore-forming microorganisms and belonged to the phyla Actinobacteria and Firmicutes, whereas culture-independent techniques identified sequenced clones affiliated with members of the phyla Actinobacteria and Proteobacteria. The diversity of fungi was shown to be much lower than that observed for bacteria, and only species of *Penicillium* spp. could be detected by cultivation techniques. The selected strategy revealed a higher microbial diversity on the obverse than on the reverse side of the painting and the near absence of actively growing microorganisms on areas showing no visible damage. Furthermore, enzymatic activity tests revealed that the most widespread activities involved in biodeterioration were esterase and esterase lipase among the isolated bacterial strains, and esterase and *N*-acetyl-β-glucosaminidase among fungi strains.

## Introduction

Nowadays, it is well known that microorganisms may be responsible for the deterioration of artefacts of cultural heritage. Two main factors responsible for the proliferation of microorganisms on art objects, especially on paintings, are: the chemical nature of the substratum and the environmental conditions, such as the availability of nutrients and favourable humidity (Saiz-Jiménez 1993). For instance, fungi are able to grow in an environment with a relative humidity higher than 65 % and temperatures ranging between 20 and 35 °C (Garg et al. 1995).

Microbial-induced deterioration (MID) effects on paintings can occur on both the obverse and the reverse side. The degree of deterioration on the obverse side depends on the paint medium (oil paints, distemper or watercolours) and mode of application, while on the reverse side, it depends on the nature of the support (canvas or wood) (Tiano 2002). In the case of oil paintings on canvas, the biodeterioration process usually starts on the reverse side of the painting due to the presence of support polymers and the glue sizing in the canvas; these components can act as substrates for microbial growth (Tiano 2002). On the other hand, the organic materials present on the obverse of the painting are susceptible to attack by specialized microorganisms and by occasional contaminants, such as transient airborne microorganisms. Bacteria and fungi represent an important part of this subaerial community which can accumulate on the painted surface for a long time as spores. The further growth of these deposited microorganisms can result in the detachment of the paint layer from the support, especially in paintings kept under conditions of high humidity (Ciferri 1999; Schabereiter-Gurtner et al. 2001a). Moreover, the excretion of aggressive metabolic products (organic or inorganic acids) and the additional production of extracellular enzymes increase the loss of material (Ciferri 1999). The main enzymatic activities involved in the deterioration of paintings caused by microorganisms are due to lipases, which catalyse hydrolysis of ester bonds of triacylglycerols at the interface between an insoluble substrate and water (Soliman et al. 2007), esterases, which act only on water-soluble substrates (Soliman et al. 2007), endo-*N*-acetyl-glucosaminidases (ENGases) acting on murein or chitin (Karamanos 1997) and proteases.

For the correct conservation and restoration of biodeteriorated art works, a detailed knowledge of the microbial communities associated with these substrates is pre-requisite to facilitating and conceiving the most suitable preservation and restoration strategies (Schabereiter-Gurtner et al. 2001a; Suihko et al. 2007; Capodicasa et al. 2010). In this regard, there are many studies reporting on the microbial diversity associated with the biodeterioration of mural paintings and frescoes (Ciferri 1999; Schabereiter-Gurtner et al. 2001a; Piñar et al. 2001; González and Saiz-Jiménez 2005). However, few studies have been published describing the microbial communities dwelling on canvas or wood-panel paintings (Capodicasa et al. 2010). Therefore, the objective of this study was to investigate the microbial communities adhering to the painted surface and the reverse side of an oil painting on canvas, and to elucidate their degradative capabilities. To this end, samples were taken from areas where visual inspection revealed signs of biodeterioration, such as changes in the colours of paints, appearance of stains or variations in the structure of the painted layer. Samples showing no visual damage were taken as controls. In addition, air samples were analysed in order to investigate the indoor air quality. A strategy combining culture-dependent and culture-independent techniques was chosen for their complementary aspects (Laiz et al. 2003).

## Materials and methods

### Sampling

**Fig. 1:**
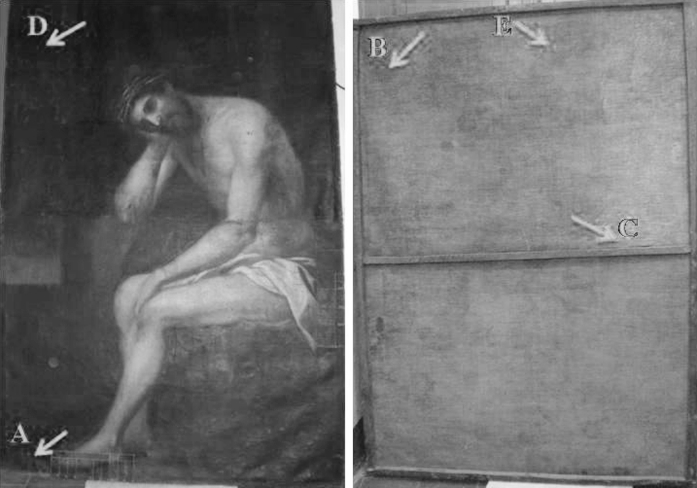
The painting ‘Cristo de la Paciencia’ (oil on canvas) and the sampling areas. **a** Sample CP1 and CP7, obverse side of the painting in an area showing signs of biodeterioration. **b** Sample CP2, reverse side in an area showing signs of biodeterioration. **c** Sample CP3, reverse side in areas showing no signs of biodeterioration. **d** Sample CP5, face (obverse) side in an area showing no signs of biodeterioration. **e** Sample CP6, reverse side in an area showing signs of biodeterioration

The oil painting on canvas ‘Cristo de la Paciencia’, which showed biodeterioration signs on both the face and the reverse side (Fig. 1), was selected for this investigation. The painting, by an anonymous artist, belongs to the Baroque period (17th–18th Centuries) and was exhibited at the convent of San Antón (Granada, Spain), which has a history of problems related to wall humidity. Since San Antón is a cloistered convent, permission to conduct a continuous measurement of the exact relative humidity and temperature inside the convent was not granted, but the yearly RH ranged from 50 to 60 % and the temperature from 8 to14 °C in winter and 20 to 24 °C in summer, respectively. These measurements were recorded inside a room located outside the entrance to the cloister.

During a restoration campaign, two types of samples were collected to characterize the microbial community present on the painting and on the indoor environment, these being: swab samples from the painting and air samples collected from the air of the room in which the painting was exhibited.

Four samples were taken from the painting by a non-invasive sampling procedure (Pinzari et al. 2009) of rubbing a sterile and dry cotton bud on the surface of the painting over an area of 2 cm^2^. For biological sampling, we used swabs (sterilized by ethylene oxide and individually wrapped in peel-pack) deemed suitable for isolations in culture media (Class IIa) (Eurotubo, Deltalab, Rubí, Spain). Two of the samples were collected from areas showing colour changes and the appearance of stains: sample CP7 from the face and CP6 from the reverse side. The remaining two samples were collected from areas showing no visible damage and were used as controls: sample CP5 from the face and CP3 from the reverse side. Cultivation assays were performed immediately on these samples.

Two additional samples (CP1, obverse and CP2, reverse) were collected from areas showing very intense signs of biodeterioration. In this case, the restorers decided to use invasive sampling by scraping off the biofilm on the surface of the canvas using sterile surgical scalpels (Bayha GmbH, Germany) and vials (sterile Eppendorf tubes, Eppendorf AG, Germany). The use of a destructive method is permissible only on fragments that cannot undergo conservation or cannot be reunited (i.e. fragments from the margins and reverse sides, bore dust produced by insects, biofilms and parts that will certainly be eliminated during restoration). The amount of material scraped off was ~0.05 g from each sample. The scrapings were stored at −80 °C for molecular assays.

In order to investigate the presence of airborne microorganisms on the indoor environment of the convent, air samples were taken and analysed both quantitatively and qualitatively using a Microbio air sampler (F.W. Parrett Limited, London, UK). Airborne microorganisms were collected by drawing a stream of air at a constant flow rate of 100 l/minute, through a series of 1 mm holes in a metal head, onto a sterile culture medium in a 90 mm Petri dish. After exposure to the air stream, the contact plate was removed and incubated. The number of colony-forming units (CFUs) which developed was counted, enabling a calculation to be made of the concentration of microorganisms in the air (cfu/m^3^-colony-forming units per cubic metre). A count correction was performed based on the sampling head used on the MicroBio air sampler. These calculations were performed automatically in the PC reporter software. Air sampling was conducted for 2 min, collecting a total of 200 l.

### Cultivation strategy

Microorganisms collected from air samples were isolated upon Trypticase soy agar (TSA, Scharlau Chemie S.A., Barcelona, Spain) and Sabouraud-chloramphenicol agar medium (Scharlau). TSA plates were incubated at 28 °C for 48 h, whereas Sabouraud-chloramphenicol plates were incubated at 28 °C for 7 days. Fungal CFUs were counted on Sabouraud-chloramphenicol agar plates, and the total number of CFUs (bacterial and fungal CFUs) was counted on TSA plates. Colonies showing different morphology and appearance were transferred to new culture plates of TSA medium for bacteria and potato dextrose agar (PDA, Scharlau) for fungi to obtain pure cultures.

Cotton swabs were inoculated directly onto agar plates containing TSA and Sabouraud-chloramphenicol agar medium and were incubated under the following conditions: 28 °C over a total period of 2 weeks. During this period, colonies exhibiting different morphology and appearance were transferred to new culture plates of TSA medium for bacteria and potato dextrose agar (PDA) for fungi to obtain pure strains. All purified strains were stored in 70 % glycerol at −80 °C for conservation.

### Genotyping of pure strains by random amplified polymorphic DNA (RAPD) analysis

Genomic DNA from pure bacterial strains was extracted following the protocol described by Ausubel et al. (1991), which relies on a chemical disruption of cells (briefly: cells were lysed and proteins removed by digestion with proteinase K. Cell wall debris, polysaccharides and remaining proteins were removed by selective precipitation with CTAB and high molecular weight DNA recovered by ethanol precipitation). DNA extraction of pure fungal strains was performed as previously described by Sert and Sterflinger (2010), with slight modifications. Briefly: samples were placed in a 1.5-ml bead beater tube with 0.4 g glass beads (0.75–1 mm, Carl Roth GmbH Co.KG, Karlsruhe, Germany) and 500 μl extraction buffer I [50 mM Tris–HCl, 150 mM NaCl, 50 mM EDTA and 0.3 % SDS (w/v), pH 8.0] and processed twice in the Fast Prep FP120 Ribolyzer (Thermo Savant Holbrook, USA) for 40 s at speed 6 m/sec. Between these ribolyzing steps, samples were incubated at 65 °C for 1 h at 800 rpm. Further DNA extraction was performed with (1:1 Vol) chloroform-isoamyl alcohol (24:1 v/v; Roth, Germany) and (1:1 Vol) phenol/chloroform/isoamyl alcohol (25:24:1, v/v; Roth, Germany), and the resultant DNA precipitated in ethanol.

For RAPD analyses, PCRs were executed in an MJ Research PTC-200 Peltier Thermal Cycler using a PCR Master Mix (Promega, Mannheim, Germany). For PCRs , 2 × PCR-MasterMix from Promega [50 units/ml of TaqDNA Polymerase in a supplied reaction buffer (pH 8.5), 400 μM dATP, 400 μMdGTP,400 μM dCTP, 400 μMdTTP, 3 mMMgCl2] was diluted to 1×, and 50 pmol/μl of each primer (VBC-Biotech, Vienna, Austria) was applied to the reaction volumes. PCRs were carried out in 25 and 2.5 μl of DNA template was added. For RAPD analysis of bacterial strains, the primer D11344 (Ripka et al. 2006) was used under the thermocycling conditions described by Welsh and McClelland (1990). For RAPD analysis of fungal strains, PCR was performed using the primer PELF (Hong et al. 2005) under the same thermocycling conditions. Gel electrophoresis was carried out in a 2 % (w/v) agarose gel for 160 min at 70 V, stained in 1 μg/ml ethidium bromide solution for 30 min and visualized by a UVP documentation system (BioRad Transilluminator, Universal Hood; Mitsubishi P93D-printer).

### DNA extraction from canvas material and PCR analysis

Total DNA from the canvas material was directly extracted from the scraping of the painting, according to the protocol described by Schabereiter-Gurtner et al. (2001a), which relies on the combination of chemical and mechanical disruption of cells and a further DNA purification by using the QIAamp Viral RNA Mini Kit (QIAGEN GmbH, Hilden, Germany), with the following modification adapted for this material: all volumes of the reagents were duplicated to compensate for the loss of volume absorbed by the canvas.

All PCRs were executed as described above, by means of the MJ Research PTC-200 Peltier Thermal Cycler and a PCR Master Mix from Promega (Mannheim, Germany). PCRs were carried out in 25 and 2.5 μl of DNA template was added.

PCR amplification of bacterial and fungal DNA directly extracted from the canvas material was performed as follows. For the amplification of bacterial 16S rDNA fragments, primer pair 341f/985r was used under the following thermocycling conditions: 5 min denaturation at 95 °C, followed by 30 cycles consisting of 1 min denaturation at 95 °C, 1 min primer annealing at 55 °C and 1 min primer extension at 72 °C, followed by a final extension step of 5 min at 72 °C. For genetic fingerprints, a semi-nested PCR was performed in a total volume of 50 μl with primers 341f-GC and 518r (Muyzer et al. 1993) using the same PCR conditions as Ettenauer et al. (2010).

For the amplification of fungal ITS regions, fragments of 450–600 bp in size, corresponding to the ITS1 and the ITS2 regions, and the 5.8S rRNA gene situated between them, were amplified with the primer pairs ITS1 forward and ITS4 reverse (White et al. 1990). The thermocycling programme was as follows: 5 min denaturation at 95 °C, followed by 35 cycles of 1 min denaturation at 95 °C and 1 min annealing at 55 °C, and 1 min extension at 72 °C. 5 min at 72 °C was performed as a final extension step. No genetic fingerprints were performed with fungal DNA.

### DGGE analysis

For DGGE analyses of bacterial communities, 100 μl PCR products, obtained as described above, were precipitated with 96 % ethanol at −20 °C overnight and re-suspended in 20 μl double-distilled H_2_O. Gel electrophoresis was carried out as described by Muyzer et al. (1993) using a D GENE-System (Bio-Rad). A linear chemical gradient ranging from 30 to 55 % (100 % denaturant containing 7 M urea and 40 % v/v formamide) was used. Gel electrophoretic separation was carried out at 60 °C and 200 V for 3.5 h. Gels were stained in 1 μg/ml ethidium bromide solution for 20 min and visualized by a UVP documentation system (BioRad Transilluminator).

### Creation of bacterial clone libraries

A clone library containing the bacterial 16S rDNA fragments of sample CP1 was created as described by Ettenauer et al. (2011), but the primer 985r (reverse) (Heuer et al. 1999) was used. The screening of the clones by DGGE was performed as previously described by Schabereiter-Gurtner et al. (2001a). The clones displaying different fingerprints were selected for sequencing.

### Sequencing analysis

For sequencing analyses of pure bacterial strains derived from cotton swabs cultures, a fragment (~650 bp) of the 16S rDNA was amplified using the primer pair 341f/985r. The thermocycling programme used was the same as that described for bacteria in paragraph 2.4 of this section. The accession numbers are listed in Table 1. Bacterial strains derived from air samples were identified by sequencing 16S rRNA fragments amplified with the primer pair WO1f/WO12r (Ogier et al. 2002) following the protocol described by Martín-Platero et al. (2009). The accession numbers were KC009523 to KC009534.

**Table 1:** Identification of the bacterial strains on the basis of 16S rDNA and identification of the fungal strains on the basis of internal transcribed spacer 1 (partial sequence), 5.8S ribosomal RNA gene and internal transcribed spacer 2 (complete sequence), and 28S ribosomal RNA gene (partial sequence) sequence analysis

Representative strains from RAPD group	Isolated from the obverse or the reverse side	Phylum	Closest related strain on basis of 16S rRNA gene sequence	Similarity (%)	Accession numbers of the sequences submitted to genbank
CP5B1	Obverse	Actinobacteria	*Microbacterium sp.* [DQ658916.1; FJ169470.1; FJ715739.1; FJ588231.1]	100 %	JN808868
CP6B1	Reverse	Firmicutes	*Bacillus* sp. [FN870069.1; HM037905.1; HM027879.1; AB360809.1; AB298784.1; GGU191902.1;GQ472195.1; GQ203617.1]	99 %	JN808869
CP6B2	Reverse	Firmicutes	*Sporosarcina* sp. [FN397659.1; EF154512.1]	99 %	JN808870
CP6B3	Reverse	Firmicutes	*Bacillus psychrodurans* strain TSC11 [EU249566.1], isolated from the Etruscan tomb of Mercareccia (Tarquinia, Italy)	99 %	JN808871
CP6B4	Reverse	Firmicutes	*Bacillus* sp. MI-58a and MI-33a1 [DQ223135.1; DQ223131.1] from a limestone cave (Kartchner Caverns)	99 %	JN808872
CP7B1	Obverse	Firmicutes	*Bacillus* sp. [HM171927.1; HM100241.1; AY043084.1]	100 %	JN808873
CP7B2	Obverse	Firmicutes	*Bacillus herbersteinensis* strain CCGE2319 [EU867376.1; AJ781029]	100 %	JN808874
CP7B3	Obverse	Firmicutes	*Virgibacillus* sp. NS3012 [GQ889491.1]	99 %	JN808875
CP7B4	Obverse	Firmicutes	*Paucisalibacillus globulus*, strain B22T [AM114102.1]	99 %	JN808876
CP7B6	Obverse	Firmicutes	*Bacillus* sp. [HQ141670.1; GU188931.1; HQ256540.1; GU586306.1]	99 %	JN808877
CP7B7	Obverse	Firmicutes	*Bacillus* sp. 19496 [AJ315064.1], isolated from biodeteriorated mural paintings in the Servilia tomb (Necropolis of Carmona, Seville, Spain)	100 %	JN808878
CP7B8	Obverse	Actinobacteria	*Arthrobacter agilis* strain 234 [EU730943.1]	100 %	JN808879
CP7B9	Obverse	Firmicutes	*Sporosarcina* sp. [FN397659.1; EU267324.1]	100 %	JN808880
CP7B10	Obverse	Firmicutes	Uncultured *Sporosarcina* sp. clone [EF074398.1]	99 %	JN808881
CP7B11	Obverse	Firmicutes	*Paucisalibacillus globulus*, strain type B22T [AM114102.1]	99 %	JN808882
CP7H3	Obverse	Ascomycota	*Penicillium* sp. [EU833227.1; EU833224.1; AY371616.1; AJ004896.1]	100 %	JN808883
CP7H4	Obverse	Ascomycota	*Penicillium* sp. [HM366606.1; GU733711.1; GQ305305.1; FJ791137.1; AJ608949.1]	97 %	JN808884

For sequencing analyses of pure fungal strains (derived from cotton swabs and air samples), fragments of about 450–600 bp in size, corresponding to the ITS1, ITS2 regions and the 5.8S rRNA gene situated between them, were amplified as described for fungi in paragraph 2.4 of this section. The accession numbers of the fungal strains isolated from cotton swabs cultures are listed in Table 1. The accession numbers of the fungal strains isolated from air samples were KC009535 to KC009539.

For sequencing of clone inserts, 100 μl PCR products were generated with primers SP6 and T7, purified using the QIAquick PCR Purification Kit (Qiagen, Hilden, Germany) and sequenced as described by Ettenauer et al. (2011). Comparative sequence analyses were performed using the BLAST search programme (Altschul et al. 1997). The sequences derived from bacterial and fungal strains and from cloned inserts were deposited at the EMBL database under the accession numbers listed in Table 1 (pure strains) and Table 2 (cloned sequences).

**Table 2:** Sequence similarities of 16S rRNA gene library clones obtained from the total DNA of sample CP1

Clon	Phylum	Length	Closest identified phylogenetic relatives [EMBL accession numbers]	Similarity (%)	Accession numbers of the sequences submitted to genbank (ID: BA123456)
CCPB2	Proteobacteria	609 bp	Uncultured *Aquabacterium* sp. clone 16S ribosomal RNA gene, partial sequence [FJ890906.1; AY569280.1]	96 %	JN808885
CCPB11	Proteobacteria	611 bp	*Citrobacter* sp. 16S ribosomal RNA gene, partial sequence [AB548828.1; AF025367.1; DQ223882.1]	96 %	JN808886
CCPB14	Proteobacteria	609 bp	*Stenotrophomonas* sp. 16S ribosomal RNA gene, partial sequence [FJ638292.1; GU254017.1; GQ417327.1; FM213389.1; EU564819.1; EU340025.1]	99 %	JN808887
CCPB17	Actinobacteria	583 bp	*Arthrobacter* sp. 16S ribosomal RNA gene, partial sequence [FJ378036.1; EU862291.1; EF110913.1; DQ310475.1]	99 %	JN808888
CCPB18	Proteobacteria	610 bp	*Providencia* sp. 16S ribosomal RNA gene, partial sequence [EU660370.1; GU193984.1; GQ417505.1]	99 %	JN808889
CCPB23	Proteobacteria	611 bp	*Stenotrophomonas* sp. 16S ribosomal RNA gene, partial sequence [FJ638292.1; GU254017.1; GQ417327.1; FM213389.1; EU564819.1; EU340025.1]	99 %	JN808890
CCPB25	Proteobacteria	609 bp	*Delftia* sp. 16S ribosomal RNA gene, partial sequence [FJ594443.1; EU707799.1; CP000884.1; AM180725.1; AY367028.1; AF538930.1]	100 %	JN808891
CCPB28	Actinobacteria	594 bp	Uncultured *Arthrobacter* sp. clone P4 s-190 16S ribosomal RNA gene, partial sequence [GQ329251.1]	99 %	JN808892
CCPB31	Proteobacteria	448 bp	Uncultured *Acinetobacter* sp. clone GI3-M-9-D06 16S ribosomal RNA gene, partial sequence [FJ191657.1]	98 %	JN808893

### Enzymatic characterization

To elucidate whether the microorganisms isolated in this study had the metabolic and destructive capabilities to be responsible for the biological degradation currently observed or even the potential for future biodeterioration of the investigated painting, an enzymatic characterization of the isolated bacterial and fungal strains was performed by using the API ZYM^®^ system (bioMérieux, France) and following the protocol of the manufacturer. This system consists of a plastic gallery of cupules carrying the enzyme substrates and allows detection of the activity of 19 enzymes: alkaline and acid phosphatases, esterase (C4), esterase lipase (C8), lipase (C14), leucine, valine and cystine aminopeptidases, trypsin, α-chymotrypsin, naphthol-AS-BI-phosphohidrolase, α-galactosidase, β-galactosidase, β-glucuronidase, α-glucosidase, β-glucosidase, β-glucosaminidase, α-mannosidase and α-fucosidase.

## Results

### Investigation of air samples

Results derived from air samples showed a concentration of between 200 and 500 cfu/m^3^ on Sabouraud-chloramphenicol and TSA media. Of the 39 different fungal strains isolated, 46.2 % were phylogenetically identified as *Penicillium* spp. (KC009535). In addition, species of the genera *Alternaria* (KC009536), *Ulocladium* (KC009537), *Stachybotrys* (KC009538) and *Cladosporium* (KC009539) were also identified on air samples. Species of other three genera could be only identified by morphological features (due to failure in the DNA extraction or further PCR amplification) and were related to *Mucor* sp., *Aspergillus* sp. and *Eurotium* sp.

Among bacteria, most of the strains isolated from air samples were identified as Gram + bacilli. Comparative sequence analyses revealed similarity values ranging from 97 to 100 % with sequences from the NCBI database and showed that these strains were related to the phylum Firmicutes, namely to the genera *Bacillus* (KC009525- KC009529 and KC009530- KC009534) and *Planomicrobium* (KC009523). In addition, two strains identified as member of the phylum Actinobacteria, belonging to the genera *Kocuria* (KC009524) and *Nocardia* (KC009529), and two further strains identified as Gram + cocci, were detected.

### Identification of the cultivable members of the microbial community colonizing the painting

Swab samples collected from areas with visible deterioration tested positive for cultivable fungi and bacteria. Eleven bacterial and two fungal strains, differing in morphology and appearance, were isolated from sample CP7 (collected from the obverse side). From sample CP6 (reverse side), only four bacterial strains were isolated and no fungal strains could be cultivated. Control samples, collected from areas showing no visible damage, yielded different results: only one bacterial strain could be isolated from sample CP5 (obverse side) and neither bacterial nor fungal specimens could be isolated from sample CP3 (reverse side).

**Fig. 2:**
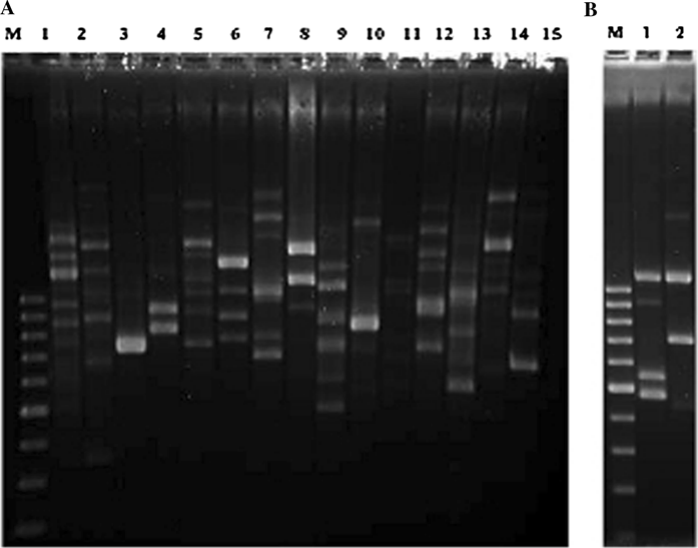
RAPD profiles derived from one representative bacterial and fungal strain of each RAPD group. **a** Bacterial strains. Lane M: 100 bp ladder; lane 1: strain CP6B1; lane 2: strain CP6B2; lane 3: strain CP6B3; lane 4: strain CP6B4; lane 5: strain CP7B1; lane 6: strain CP7B2; lane 7: strain CP7B3; lane 8: CP7B4; lane 9: strain CP7B6; lane 10: strain CP7B7; lane 11: strain CP7B8; lane 12: strain CP7B9; lane 13: strain CP7B10; lane 14: strain CP7B11; lane 15: strain CP5B1. **b** Fungal strains. Lane M: 100 bp ladder; lane 1: CP7H1; lane 2: CP7H2

The genetic diversity of the bacterial and fungal isolates was evaluated by RAPD analysis (Fig. 2). Based on RAPD profiles, bacterial isolates could be clustered into 15 different groups (Fig. 2a) and fungal strains into two groups (Fig. 2b). One representative strain of each RAPD cluster was selected for sequencing and identification. These sequences were compared with sequences deposited in the EMBL database (GenBank). The identification of the bacterial and fungal strains is presented in Table 1.

The cultivable fraction of the bacterial community colonizing the obverse of the painting (sample CP7) showed a structure comprising bacteria affiliated with four genera of two phyla: *Bacillus*, *Sporosarcina*, *Paucisalibacillus* and *Virgibacillus,* belonging to the phylum Firmicutes, and *Arthrobacter,* belonging to the phylum Actinobacteria. The structure of the community colonizing the reverse side of the painting (sample CP6) consisted of bacterial strains affiliated with two genera of the phylum Firmicutes: *Bacillus* and *Sporosarcina*.

The cultivable fraction of the fungal community proved to be poor since fungi could be only isolated from the obverse side of the painting (sample CP7). Both fungal strains were affiliated with *Penicillium* sp., which belongs to the phylum Ascomycota.

### Identification of the non-cultivable members of the microbial community colonizing the painting

To investigate the non-cultivable fraction of the microbial community associated with the painting material, total DNA was directly extracted from samples CP1 and CP2, collected from the face and the reverse side of the painting, respectively, and used as a template for PCR analysis using bacterial and fungal-specific primers. The amplification of total DNA from sample CP1 showed positive results using bacterial primers, whereas fungal-specific primers yielded no DNA amplification (data not shown). On the other hand, the extraction and further amplification of total DNA from sample CP2 yielded negative results when using bacterial and fungal primers, indicating an absence or lower concentration of microorganisms (below the detection level of the techniques used in this study) on the reverse side of the painting.

**Fig. 3:**
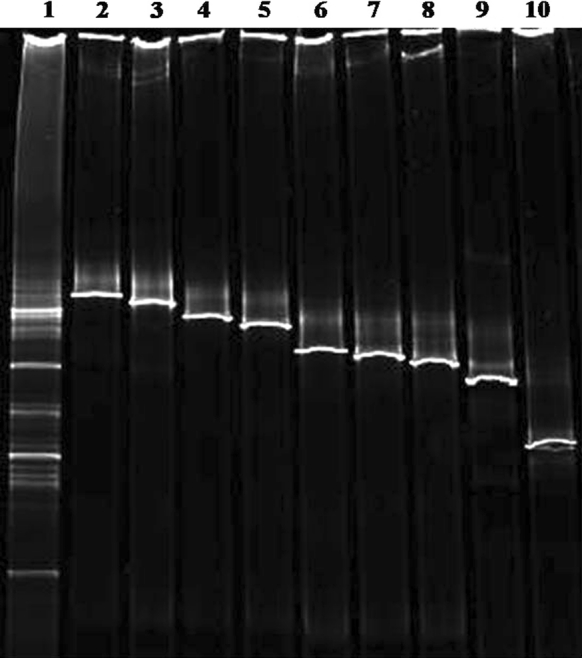
DGGE profiles of clones containing 16S rDNA bacterial fragments obtained from sample CP1 and producing PCR products with different motility behaviour. Lane 1: bacterial community fingerprint of sample CP1; lane 2: CCPB2; lane 3: CCPB11; lane 4: CCPB14; lane 5: CCPB17; lane 6: CCPB18; lane 7: CCPB23; lane 8: CCPB25; lane 9: CCPB28; lane 10: CCPB31

The amplified bacterial DNA of sample CP1 was further analysed by DGGE fingerprinting, which showed four dominant bands as well as some other faint bands (Fig. 3). The resulting clones containing bacterial 16S rDNA fragments were screened on DGGE, and inserts of clones producing PCR products showing different motility behaviour, and which could be matched with bands from the DGGE profile of sample CP1, were selected for sequencing. Nine different clone inserts were sequenced and compared with known bacterial sequences from the EMBL database (Table 2).

Clone inserts were affiliated with species belonging to the phyla Proteobacteria and Actinobacteria. The detected Proteobacteria belonged to six different genera, namely *Aquabacterium*, *Citrobacter*, *Stenotrophomonas*, *Providencia*, *Delftia* and *Acinetobacter*. The two cloned sequences affiliated with the phylum Actinobacteria belonged to the genus *Arthrobacter*.

### Enzymatic characterization

Results derived from the enzymatic characterization of the isolated bacterial and fungal strains (Table 3) showed that the most widespread enzymatic activities related to biodeterioration were, among bacterial isolates, esterase and esterase lipase. Among fungi, both isolates share the activities esterase and *N-*acetyl-β-glucosaminidase.

**Table 3:** Enzymatic characterization of the cultivated bacteria and fungi

Enzymatic Activity	Bacterial Isolates														Fungal Isolates
	*Microbacterium* sp.	*Bacillus* sp.					*Sporosarcina* sp.			*Bacillus psychrodurans*	*Bacillus herbersteinensis*	*Virgibacillus* sp.	*Paucisalibacillus globulus*	*Arthrobacter agilis*	*Penicillium* sp.
	CP4B1	CP5B1	CP5B4	CP6B1	CP6B6	CP6B7	CP6B2	CP6B9	CP6B10	*s* CP5B3	CP6B2	CP6B3 ;CP6B5	CP6B4; CP6B11	CP6B8	CP7H3; CP7H4
Phosphatase alkaline	**−**	**−**	**−**	**+**	**−**	**−**	**−**	**−**	**−**	**−**	**−**	**−**	**+**	**−**	**+**
Esterase (C4)	+	**+**	**+**	**+**	**−**	**+**	**−**	**−**	**+**	**+**	**−**	**+**	**+**	**+**	**+**
Esterase Lipase (C8)	**−**	**+**	**+**	**−**	**+**	**+**	**−**	**−**	**−**	**−**	**−**	**+**	**+**	**+**	**−**
Lipase (C14)	**−**	**−**	**−**	**−**	**+**	**−**	**−**	**−**	**−**	**−**	**−**	**−**	**−**	**−**	**−**
Leucine aminopeptidase	**−**	**−**	**−**	**−**	**−**	**−**	**+**	**−**	**−**	**−**	**−**	**−**	**−**	**−**	**−**
Valine aminopeptidase	**−**	**−**	**−**	**−**	**−**	**−**	**−**	**−**	**−**	**−**	**−**	**−**	**−**	**−**	**−**
Cystine aminopeptidase	**−**	**−**	**−**	**−**	**−**	**−**	**−**	**−**	**−**	**−**	**−**	**−**	**−**	**−**	**−**
Trypsin	**−**	**−**	**−**	**−**	**−**	**−**	**−**	**−**	**−**	**−**	**−**	**−**	**−**	**−**	**−**
α−Chymotrypsin	**−**	**−**	**−**	**−**	**−**	**−**	**−**	**−**	**−**	**−**	**−**	**−**	**−**	**−**	**−**
Phosphatase acid	**−**	**−**	**+**	**−**	**−**	**+**	**−**	**−**	**+**	**−**	**−**	**−**	**−**	**−**	**+**
Naphthol-AS-BI-															
Phosphohydrolase	**+**	**+**	**+**	**+**	**+**	**+**	**+**	**+**	**+**	**+**	**+**	**+**	**+**	**+**	**+**
α-Galactosidase	**−**	**−**	**−**	**+**	**−**	**−**	**−**	**+**	**−**	**−**	**+**	**−**	**−**	**+**	**−**
β-Galactosidase	**−**	**+**	**−**	**+**	**−**	**−**	**−**	**+**	**−**	**−**	**+**	**−**	**+**	**+**	**−**
β-Glucuronidase	**−**	**−**	**−**	**−**	**−**	**−**	**−**	**−**	**−**	**−**	**−**	**−**	**−**	**−**	**−**
α-Glucosidase	**−**	**−**	**−**	**+**	**+**	**−**	**−**	**+**	**+**	**+**	**+**	**−**	**+**	**+**	**−**
β-Glucosidase	**−**	**+**	**−**	**−**	**−**	**−**	**−**	**−**	**−**	**−**	**−**	**−**	**−**	**−**	**−**
*N*-Acetyl-β-glucosaminidase	**−**	**−**	**−**	**−**	**−**	**−**	**−**	**−**	**−**	**−**	**−**	**−**	**−**	**−**	**+**
α-Mannosidase	**−**	**−**	**−**	**−**	**−**	**−**	**−**	**−**	**−**	**−**	**−**	**−**	**−**	**−**	**−**
α-Fucosidase	**−**	**−**	**−**	**−**	**−**	**−**	**−**	**−**	**−**	**−**	**−**	**−**	**−**	**−**	**−**

(+): detectable and (-): non-detectable enzymatic activity

## Discussion

### Comparison of the microbial communities found on the obverse and the reverse side of the painting and in the air

As we explained previously in the introduction, the biodeterioration process on paintings usually starts on the reverse side due to the composition of the substrates present on the canvas, which are more readily degraded than those found on the obverse side (Tiano 2002). Nevertheless, the results obtained in this study showed a higher microbial density and complexity on the obverse side of the investigated painting. Results derived from cultivation assays were further supported by data obtained from culture-independent techniques: DNA fingerprints could be obtained only from sample CP1, taken from the obverse of the painting, demonstrating the higher amount of microorganisms on this side of the painting. This fact may be related to the poorly controlled convent environment. The indoor air was laden with airborne, spore-forming microorganisms which showed to be a reflection of the composition of the settled dust collected from the painted surface. The microorganisms identified on air samples correlated with those isolated from the surface of the painting, when non-invasive sampling was used; in both cases, the most frequently isolated strains belonged to spore-forming microorganisms, such as *Bacillus*-related species or *Penicillium* sp. The similarity in the microbial community (fungi and bacteria) found on the surface of the painting and in the air samples may indicate that the likely source of microbial contamination was the gravitational settling of spores from the air onto the painting, and therefore, the spores found on the surfaces may reflect the airborne microbial community found in that indoor environment (Hyvärinen 2002).

The spore-forming bacteria detected in this study have been frequently isolated from various cultural heritage sites such as mural paintings (Gorbushina et al. 2004; Pepe et al. 2010), panel paintings (Capodicasa et al. 2010), stained glass (Marvasi et al. 2009) or paper materials (Michaelsen et al. 2010). Moreover, the ability of members of the genus *Bacillus* to transform into spores allows them to maintain viability in unfavourable, dry conditions for a long period of time. Therefore, species of this genus showed to be the dominant bacteria settled on the painted surface (Karbowska-Berent et al. 2011).

The *Penicillium* sp. detected in this study have been also described in articles considering different works of art: mural and panel paintings (Piñar et al. 2009; Capodicasa et al. 2010; Pepe et al. 2010), the interior of stone monuments (Suihko et al. 2007), canvas (Vukojević and Grbić 2010) and paper (Michaelsen et al. 2010). The genus *Penicillium* has been reported as the most frequently found fungal genus in indoor air and buildings (Hyvärinen 2002). The fact that dry spores of *Penicillium* sp. are more easily released into air than spores of other fungal genera, due to the production of conidia which allow them to produce a high number of spores (Pasanen et al. 1991), may partly explain the frequent occurrence of *Penicillium* sp. in indoor air (Burge et al. 2000).

In addition, cultivation assays evidenced differences between samples taken from areas showing biodeterioration and control areas with no visible damage. Only one bacterial strain belonging to the Actinobacteria could be isolated from sample CP5 (obverse), and negative cultivation results were obtained from sample CP3 (reverse).

### Comparison of results derived from culture-dependent and culture-independent techniques

Depending on the methodology used, some differences with regard to the community structure detected were observed (see Table 1 vs. Table 2). Culture-dependent techniques allowed the detection of different bacterial species with a predominance of spore-forming bacteria, principally *Bacillus*-related species, as previously observed by other authors (Laiz et al. 2002). However, no clones harbouring sequences of members of the phylum Firmicutes could be screened from the clone library, indicating possible limitations in the extraction of DNA from spores, as reported in previous works (Laiz et al. 2003). A plausible explanation could be the low relative abundance of sequences originating from vegetative cells in the initial bacterial community, indicating that isolates of the phylum Firmicutes are present on the painting as metabolically inactive spores.

On the contrary, strains belonging to the phylum Proteobacteria could not be isolated using cultivation methods, but were shown to be dominant when total DNA was extracted directly. This fact can be due to the introduction of this group of bacteria at a viable, but non-cultivable stage (VBNC), since it has already been reported that dormant microorganisms are unable to grow on standard culture media (Roszak and Colwell 1987). However, we cannot rule out the possibility of the amplification of free DNA from dead microorganisms. Only species of *Arthrobacter* sp. were identified by both techniques.

Species of the genera *Arthrobacter*, *Acinetobacter*, *Stenotrophomonas* and *Delftia* have been identified in previous studies examining different art works (Gorbushina et al. 2004; Piñar et al. 2009, 2010; Santos et al. 2009; Capodicasa et al. 2010; Jroundi et al. 2010; Michaelsen et al. 2010; Ettenauer et al. 2011). These results confirm that members of these genera are widespread and are an important part of the microbial communities inhabiting paintings and cultural artefacts. The remaining three clones (CCPB2, CCPB11 and CCPB18) related to species of the genera *Aquabacterium*, *Citrobacter* and *Providencia* showed no correlation with bacterial strains detected in other studies related to biodeteriorated works of art.

Concerning the fungal community, no fungal DNA could be amplified from DNA extracted from sample CP1; however, two fungal strains belonging to *Penicillium* sp. could be isolated from that area using culture-dependent methods. This is most probably due to the low proportion of fungi in the microbial community, but it could be also due to a low efficiency of the DNA extraction method from spores, which could indicate that fungi associated with this painting exist only as spores and, consequently, they were metabolically inactive at the time of sampling.

### Evaluation of the potential risk for the painting

Our data suggest that a high proportion of the microbial community was inactive at the time of sampling. As a consequence, the microorganisms detected in the painting may not be responsible for its damage since mechanisms of biodeterioration occur when microbes are metabolically active and growing. However, the presence of an actively growing microbial community in areas showing signs of biodeterioration and the absence of this community in undamaged areas may indicate the possible involvement of certain members of the community, probably those microorganisms detected by both techniques, in the deterioration of the painting. On the other hand, the dampness observed in the wall may have been responsible for meeting the humidity requirements necessary to promote bacterial and fungal growth (Garg et al. 1995). In that case, environmental conditions favourable for microorganisms may have existed for some time in the convent investigated, allowing the development of a biofilm (Gorbushina et al. 2004). Indeed, the higher complexity of the microbial communities observed on the obverse of the painting could be a consequence of the high amount of airborne microorganisms accumulating on the painted surface. It is worth remarking that a wide representation of spore-formers among bacterial as well as fungal isolates reflects the adaptation of a subaerial system (microbial growth on atmosphere-exposed surfaces) to indoor environments, which offer quite stable environments (Roszak and Colwell 1987; Gorbushina et al. 2004). The detection of airborne bacteria and fungi, which can be considered merely as transient microorganisms and not the ones inhabiting and deteriorating the painting, highlights the necessity to clarify their role in the biodeterioration process (Schabereiter-Gurtner et al. 2001b). Only a few members of the microbial community detected in art works may grow and damage them; usually those which can resist unfavourable environmental conditions and have high enzymatic activity and low nutritional requirements (Abrusci 2005). These requirements coincide with the physiological characteristics of some microorganisms detected in our study. Results derived from the enzymatic characterization (Table 3) showed that the most widespread bacterial enzymatic activities related to biodeterioration were esterase and esterase lipase, both of which are involved in the hydrolysis of lipids, and therefore, have the potential to degrade painting constituents such as linseed oil. In addition to esterase activity, fungi showed *N-*acetyl-β-glucosaminidase activity, which is capable of hydrolysing the alternative linkage in the polysaccharide backbone of the bacterial cell wall peptidoglycan. This, in conjunction with other enzymatic activities, could convert the peptidoglycan to oxidizable mono saccharides when no nutrients are available (Priest 1977). Thus, this enzymatic characterization demonstrated that most fungi and bacteria isolated in this study displayed the necessary metabolic activities to be responsible for the biological attack observed on the painting.

It can be assumed that the deterioration of the painting ‘Cristo de la Paciencia’ is caused more by the environmental conditions found in the convent than by the chemical nature of the substrate itself. The presence of spores on the surface of the painting invites the risk of future biodeterioration if changes in the environmental conditions allow spores to germinate. This scenario will prevail for as long as the environmental parameters are not maintained under controlled conditions in the convent.

## Conclusions

The microbial communities adhering to the painted surface and the reverse side of an oil painting on canvas, hanging in a poorly controlled convent environment, were investigated by culture-dependent and culture-independent techniques. Results derived from both techniques revealed a higher microbial complexity on areas displaying biodeterioration. Furthermore, microorganisms were shown to be dominant on the obverse side of the investigated painting, whereas on the reverse side, microorganisms were, in some cases, beneath the detection limit of the techniques used in this study. This fact was shown to be directly related to the indoor quality air of the room where the painting was exposed. The air was laden with airborne microorganisms which could adhere to the surface of the painting.

However, differences in the community structure could be observed when using different techniques. Culture assays revealed the dominance of airborne spore-forming bacteria, members of the Firmicutes, whereas molecular techniques showed the dominance of more specialized microorganisms, members of the Proteobacteria. Only members of the Actinobacteria, namely species of *Arthrobacter*, were identified by both techniques.

In addition, the enzymatic characterization of the strains isolated in this study displayed different activities with potential for the destruction of the investigated material. Moreover, we detected the presence of sequences related to species of the genera *Acinetobacter*, *Stenotrophomonas* and *Delftia*, which have been identified previously in other studies to be responsible for the biodeterioration of other works of art. Our results confirm that members of these genera are widespread and an important part of the microbial communities inhabiting paintings and cultural artefacts.

## References

[CR1] Abrusci C (2005). Isolation and identification of bacteria and fungi from cinematographic films. International Biodeterioration and Biodegradation.

[CR2] Altschul SF, Madden TL, Schäffer AA, Zhang J, Zhang Z, Miller W, Lipman JD (1997). Gapped BLAST and PSI-BLAST: A new generation of protein database search programs. Nucleic Acids Research.

[CR3] Ausubel FM, Brent R, Kingston RE, Moore DD, Seidman JG, Smith JA, Struhl K (1991). Current Protocols in Molecular Biology.

[CR4] Burge HA, Pierson DL, Groves TO, Strawn KF, Mishra SK (2000). Dynamics of airborne fungal populations in a large office building. Current Microbiology.

[CR5] Capodicasa S, Fedia S, Porcellia AM, Zannoni D (2010). The microbial community dwelling on a biodeteriorated 16th century painting. International Biodeterioration and Biodegradation.

[CR6] Ciferri O (1999). Microbial degradation of paintings. Applied and Environmental Microbiology.

[CR7] Ettenauer J, Piñar G, Sterflinger K, Gonzalez-Muñoz MT, Jroundi F (2011). Molecular monitoring of the microbial dynamics occurring on historical limestone buildings during and after the in situ application of different bio-consolidation treatments. Science of the Total Environment.

[CR8] Ettenauer J, Sterflinger K, Piñar G (2010). Cultivation and molecular monitoring of halophilic microorganisms inhabiting an extreme environment presented by a salt-attacked monument. International Journal of Astrobiology.

[CR9] Garg KL, Jain KK, Mishra AK (1995). Role of fungi in the deterioration of wall paintings. Science of the Total Environment.

[CR10] González JM, Saiz-Jiménez C (2005). Application of molecular nucleic acid-based techniques for the study of microbial communities in monuments and artworks. International Microbiology.

[CR11] Gorbushina AA, Heyrman J, Dorniedena T, Gonzalez-Delvalle M, Krumbein WE, Laiz L, Petersen K, Saiz-Jimenez C, Swings J (2004). Bacterial and fungal diversity and biodeterioration problems in mural painting environments of St. Martins church (Greene–Kreiensen, Germany). International Biodeterioration and Biodegradation.

[CR12] Heuer H, Hartung K, Wieland G, Kramer I, Smalla K (1999). Polynucleotide probes that target a hypervariable region of 16S rRNA genes to identify bacterial isolates corresponding to bands of community fingerprints. Applied and Environmental Microbiology.

[CR13] Hong S-B, Go S-J, Shin H-D, Frisvad J, Samson RA (2005). Polyphasic taxonomy of *Aspergillus fumigatus* and related species. Mycologia.

[CR14] Hyvärinen, A. (2002). Characterizing moisture damaged buildings- environmental and biological monitoring. National Public Health Institute, Department of Environmental Health, Laboratory of Environmental Microbiology (Kuopio, Finland) and University of Kuopio, Department of Environmental Sciences. PhD Thesis.

[CR15] Jroundi F, Fernández-Vivas A, Rodriguez-Navarro C, Bedmar E, González Muñoz MT (2010). Bioconservation of deteriorated monumental calcarenite stone and identification of bacteria with carbonatogenic activity. Microbial Ecology.

[CR16] Karamanos Y (1997). Endo-IV-acetyl-P-D-glucosaminidases and their potential substrates: structure/function relationships. Research in Microbiology.

[CR17] Karbowska-Berent J, Górny RL, Strzelczyk AB, Wlazło A (2011). Airborne and dust borne microorganisms in selected polish libraries and archives. Building and Environment.

[CR18] Laiz L, Hermosin B, Caballero B, Saiz-Jimenez C, Galan E, Zezza F (2002). Facultatively oligotrophic bacteria in Roman mural paintings. Protection and conservation of the cultural heritage of the mediterranean cities.

[CR19] Laiz L, Piñar G, Lubitz W, Saiz-Jimenez C (2003). Monitoring the colonisation of monuments by bacteria: cultivation versus molecular methods. Environmental Microbiology.

[CR20] Martín-Platero AM, Maqueda M, Valdivia E, Purswani J, Martínez-Bueno M (2009). Polyphasic study of microbial communities of two Spanish farmhouse goats’ milk cheeses from Sierra de Aracena. Food Microbiology.

[CR21] Marvasi M, Vedovato E, Balsamo C, Macherelli A, Dei L, Mastromei G, Perito B (2009). Bacterial community analysis on the Medieval stained glass window “Natività” in the Florence Cathedral. Journal of Cultural Heritage.

[CR22] Michaelsen A, Piñar G, Pinzari F (2010). Molecular and microscopical investigation of the microflora inhabiting a deteriorated Italian manuscript dated from the thirteenth century. Microbial Ecology.

[CR23] Muyzer G, De Waal EC, Uitterlinden AG (1993). Profiling of complex microbial populations by denaturing gradient gel electrophoresis analysis of polymerase chain reaction-amplified genes coding for 16S rRNA. Applied and Environmental Microbiology.

[CR24] Ogier JC, Son O, Gruss A, Tailliez P, Delacroix-Buchet A (2002). Identification of the bacterial microflora in dairy products by temporal temperature gradient gel electrophoresis. Applied and Environmental Microbiology.

[CR25] Pasanen AL, Pasanen P, Jantunen MJ, Kalliokoski P (1991). Significance of air humidity and air velocity for fungal spore release into the air. Atmospheric Environment.

[CR26] Pepe O, Sannino L, Palomba S, Anastasio M, Blaiotta G, Villania F, Moschetti G (2010). Heterotrophic microorganisms in deteriorated medieval wall paintings in southern Italian churches. Microbiological Research.

[CR28] Piñar G, Ramos C, Rölleke S, Schabereiter-Gurtner C, Vybiral D, Lubitz W, Denner EBM (2001). Detection of indigenous *Halobacillus* populations in damaged ancient wall paintings and building materials: molecular monitoring and cultivation. Applied and Environmental Microbiology.

[CR29] Piñar G, Ripka K, Weber J, Sterflinger K (2009). The micro-biota of a sub-surface monument: The medieval chapel of St. Virgil (Vienna, Austria). International Biodeterioration and Biodegradation.

[CR27] Piñar G, Jimenez-Lopez G, Sterflinger K, Ettenauer J, Jroundi F, Fernandez Vivas A, Gonzalez-Muñoz MT (2010). Bacterial community dynamics during the application of a *Myxococcus xanthus*-inoculated culture medium used for consolidation of ornamental limestone. Microbial Ecology.

[CR30] Pinzari F, Montanari M, Michaelsen A, Piñar G (2009). Analytical protocols for the assessment of biological damage in historical documents. Coalition.

[CR31] Priest G (1977). Extracellular enzyme synthesis in the genus *Bacillus*. Bacteriological reviews.

[CR32] Ripka K, Denner EBM, Michaelsen A, Lubitz W, Piñar G (2006). Molecular characterisation of *Halobacillus* strains isolated from different medieval wall paintings and building materials in Austria. International Biodeterioration and Biodegradation.

[CR33] Roszak DB, Colwell RR (1987). Survival strategies of bacteria in the natural environments. Microbiological Reviews.

[CR34] Saiz-Jiménez C (1993). Deposition of airborne organic pollutants on historic buildings. Atmospheric Environment.

[CR35] Santos A, Cerrada A, García S, San Andrés M, Abrusci C, Marquina D (2009). Application of molecular techniques to the elucidation of the microbial community structure of antique paintings. Microbial Ecology.

[CR36] Schabereiter-Gurtner C, Piñar G, Lubitz W, Rölleke S (2001). An advanced strategy to identify bacterial communities on art objects. Journal of Microbiological Methods.

[CR37] Schabereiter-Gurtner C, Piñar G, Lubitz W, Rölleke S (2001). Analysis of fungal communities on historical church window glass by denaturing gradient gel electrophoresis and phylogenetic 18S rDNA sequence analysis. Journal of Microbiological Methods.

[CR38] Sert HB, Sterflinger K (2010). A new *Coniosporium* species from historical marble monuments. Mycological Progress.

[CR39] Soliman NA, Knoll M, Abdel-Fattah YR, Schmid RD, Lange S (2007). Molecular cloning and characterization of thermostable esterase and lipase from *Geobacillus thermoleovorans* YN isolated from desert soil in Egypt. Process Biochemistry.

[CR40] Suihko M-L, Alakomi HL, Gorbushina A, Fortune I, Marquardt J, Saarela M (2007). Characterization of aerobic bacterial and fungal microbiota on surfaces of historic Scottish monuments. Systematic and Applied Microbiology.

[CR41] Tiano, P. (2002). Biodegradation of cultural heritage: Decay mechanisms and control methods “9th ARIADNE Workshop” Historic Material and their Diagnostic”, ARCCHIP, Prague, 22 to 28 April 2002. http://www.arcchip.cz/w09/w09_tiano.pdf.

[CR42] Vukojević J, Grbić ML (2010). Moulds on paintings in Serbian fine art museums. African Journal of Microbiology Research.

[CR43] Welsh J, McClelland M (1990). Fingerprinting genomes using PCR with arbitrary primers. Nucleic Acids Research.

[CR44] White TJ, Bruns T, Lee S, Taylor J (1990). Amplification and direct sequencing of fungal ribosomal RNA genes for phylogenetics. PCR protocols: A guide to methods and applications.

